# Measurement of factors that negatively influence the outcome of quitting smoking among patients with COPD: psychometric analyses of the Try To Quit Smoking instrument

**DOI:** 10.1002/nop2.4

**Published:** 2014-09-08

**Authors:** Lena Lundh, Hassan Alinaghizadeh, Lena Törnkvist, Hans Gilljam, Maria Rosaria Galanti

**Affiliations:** ^1^ Department of Neurobiology, Care Sciences and Society (NVS)Centre for Family MedicineKarolinska InstitutetHuddinge141 83Sweden; ^2^ Department for Public Health ScienceKarolinska InstitutetStockholm171 77Sweden

**Keywords:** Clinical research, COPD, exploratory factor analyses, smoking cessation, TTQ

## Abstract

**Aims:**

To test internal consistency and factor structure of a brief instrument called Trying to Quit smoking.

**Background:**

The most effective treatment for patients with chronic obstructive pulmonary disease is to quit smoking. Constant thoughts about quitting and repeated quit attempts can generate destructive feelings and make it more difficult to quit.

**Design:**

Development and psychometric testing of the Trying to Quit smoking scale.

**Methods:**

The Trying to Quit smoking, an instrument designed to assess pressure‐filled states of mind and corresponding pressure‐relief strategies, was tested among 63 Swedish patients with chronic obstructive pulmonary disease. Among these, the psychometric properties of the instrument were analysed by Exploratory Factor Analyses.

**Results:**

Fourteen items were included in the factor analyses, loading on three factors labelled: (1) development of pressure‐filled mental states; (2) use of destructive pressure‐relief strategies; and (3) ambivalent thoughts when trying to quit smoking. These three factors accounted for more than 80% of the variance, performed well on the Kaiser‐Meyer‐Olkin (KMO) test and had high internal consistency.

## Introduction

The prevalence of Chronic Obstructive Pulmonary Disease (COPD) is usually related to tobacco smoking and a result of a cumulative exposure over decades. In western countries (or in Sweden), it has been estimated that 10% of the population may suffer from COPD and 50% of the elderly smokers (>45 years) (Lundback *et al*. 2003). Therefore, COPD is still the leading cause of morbidity and mortality (GOLD 2013). In clinical settings, there is a need for a valid instrument to predict failures during attempts to quit smoking among patients with COPD. Such an instrument may enable health professionals to tailor their support on the patients’ needs. In fact, almost half of the patients with COPD continue to smoke after the diagnosis (Tashkin & Murray 2009), with consequent worsening of symptoms and prognosis of COPD (Tashkin & Murray 2009, Vozoris & Stanbrook 2011). Failure in quitting smoking among patients with COPD may also induce feelings of frustration and powerlessness among healthcare providers (Lundh *et al*. 2006), possibly leading to professional disinvestment towards these patients.

## Background

There is a wide range of instruments that can be employed by healthcare professionals to predict the outcome of a quit attempt, exploring dimensions such as nicotine dependence (Heatherton *et al*. 1991), cigarette dependence (Etter *et al*. 2003), abstinence‐related expectancies (Hendricks *et al*. 2011), self‐efficacy beliefs regarding smoking cessation (Rash & Copeland 2008, Speak *et al*. 2013) and of smoking outcome expectancies (Rash & Copeland 2008). None of these instruments seems to fully explore the difficulties patients with COPD deal with when they try to quit smoking. Patients with COPD and especially women seem to find it harder to quit smoking than patients without this diagnosis (Tashkin & Murray 2009, Vozoris & Stanbrook 2011), although the reasons of this difference are not clear.

To increase success rates in smoking cessation among patients with COPD, programmes with different characteristics and intensity have been used. Recent systematic reviews suggested that the most effective smoking cessation strategy for this patient group would be pharmacological therapy in combination with intensive counselling (Warnier *et al*. 2013) and face‐to‐face contacts yielding higher quit rates than telephone contacts (Stead & Lancaster 2012). In primary health care, where most patients with COPD are treated, low‐intensity support in smoking cessation is usually employed, such as simple advice to quit, minimal intervention or telephone counselling, alone or in combination with medications (nicotine replacement therapy, bupropion or varenicline (Cahill *et al*. 2013). To redirect and use efficiently the limited resources of healthcare providers in outpatients’ settings, it would be very useful to identify those COPD patients who might develop pressure‐filled mental states, have high risk of failure during a quit attempt or even give up any plan to quit smoking after an unsuccessful attempt.

In a recent qualitative study conducted by our group, some patients with COPD developed pressure‐filled mental states when trying to quit smoking. These mental states include feeling fearful (worries about health), feeling criticized (from self and or others), feeling pressure (continuous thoughts about quitting) and worthlessness (e.g. being a weak person). Besides having a negative impact on mental well‐being, these feelings may constitute risk factors for loss of motivation and for giving up further quit attempts (Lundh *et al*. 2012). Low self‐efficacy (Baldwin *et al*. 2006) and feeling of guilt and shame (Arne *et al*. 2007, Wilson *et al*. 2011) seem to be common among patients with COPD. Constant thoughts about quitting and repeated quit attempts, not accompanied by confidence in success, can generate very destructive feelings and the very same decision to quit may appear unbearable (Jonsdottir & Jonsdottir 2007). To date, there is no instrument that tackles these mental processes.

The scope of this study is to test the internal consistency and factor structure of a brief instrument denominated TTQ, designed for processes that negatively influence the occurrence or the outcome of quit attempts among patients with COPD.

## Methods

### Design

A survey and psychometric approach was used to test the initial reliability and validity of the TTQ scale.

### Development of TTQ

A theoretical model was used to develop the TTQ questionnaire (Lundh *et al*. 2012). The model describes the process of trying to quit smoking in patients with COPD and the reasons behind successful and unsuccessful quit attempts, resignation and continued smoking. The model identifies as specific cue to continued smoking pressure‐filled mental states when trying to quit, their causes and strategies commonly used to achieve pressure relief. Ideally, the TTQ was meant to cover the following aspects: (I) decision about smoking; (II) development of pressure‐filled mental states; (III) strategies to manage pressure‐filled mental states; and (IV) degree of hope and meaningfulness in quitting smoking. Based on the theoretical model, three dimensions and 19 items were identified to describe the process of trying to quit smoking.

The TTQ was developed through several steps and the content and face validity were assessed both by nurses and by smokers diagnosed with COPD : (1) first, four nurses responsible for patients with pulmonary diseases (experts) were asked to judge the comprehension of each item, the relevance of the questions and of the response alternatives and whether the TTQ appeared to measure target variables, i.e. factors influencing an attempt to quit smoking. After this assessment, some statements were rephrased; (2) second, 20 smokers diagnosed with COPD were asked to rate their understanding of each TTQ item. They also rated the applicability and completeness of the questionnaire as a whole. All items except three were rated as easy to understand. These three items were rephrased; (3) third, the TTQ was pre‐tested among 20 smokers with COPD. Fifteen items were analysed separately for each dimension of the theoretical model. This analysis led to a preliminary grouping of the questionnaire items in themes following the theoretical model. Some adjustments were also made in the wording.

The final version of TTQ is a 19‐item instrument covering factors likely to describe the mental processes of COPD patients before and during a quit attempt. For the (Kottner *et al*. 2011) purpose of the present study, some questions on demographic, severity of COPD and smoking history were added to the instrument. For all items the response is given on a 4‐point Likert scale ranging from 1 (do not agree at all)–4 (do agree completely). In the final three‐factor model, four items covering decisions about smoking were excluded and two positively worded items were reversed before analysis.

### Data collection

We aimed to enrol in the study 250 consecutive smoking patients with COPD attending a regular clinical visit at Primary Healthcare Centres (PHCC). Enrolment and data collection took place in 29 primary healthcare facilities in the Stockholm County. At most PHCC, one or two nurses are in charge for nurse‐led programme for pulmonary rehabilitation. In total, 171 nurses with such responsibility were invited to take part in the study, of which 31 accepted. They were each asked to recruit five patients with a diagnosis of COPD, i.e. a total of 155 patients. The patients were eligible if they currently smoked at least weekly and were able to speak Swedish. Informed consent to inclusion in the study was provided orally during the patients’ regular consultations with the corresponding nurse from April 2011–November 2012. Prior to consent, the nurses provided a covering letter about the study and pamphlet with information about smoking cessation. In a 2‐week period, each nurse recruited a mean of 3 (range 1–5) consecutive smoking patients with COPD during the patients' regular clinic visits, a total of 102 patients. Lack of time was the only reason for some nurses not being able to recruit all expected five patients.

### Statistical analysis

If not otherwise stated, demographic and smoking history characteristics are presented as mean and standard deviation, for numerical continuous variables and as frequencies and percentages for categorical variables. Comparison of continuous variables between groups was done by a two‐tailed *t*‐test, where normality assumption was met. For categorical variables, the chi‐square test was used when expected values were more than five; otherwise, the Fisher exact test was used.

Internal consistency (Chronbach's alpha) was evaluated for reliability. The acceptable value for minimum reliability coefficient was >0·70 (Kottner *et al*. 2011). To explore the number of dimensions in TTQ, exploratory factor analyses (EFA) were conducted, using unweighted least squares (ULS) method based on polychoric correlation matrix followed by orthogonal varimax rotation. Kaiser–Meyer–Olkin (KMO) was performed to confirm adequate number of factors in data. The goodness of fit with three factors was confirmed after rotation. The Likelihood ratio test (chi‐square) was used to compare the chosen three‐factor model against the saturated factors model with conventional statistical significance indicated by *P* < 0·05. Critical selection of items in the factor analysis was: no cross‐loading; loading value over 0·30; uniqueness over 0·60. Bartlett's test of Sphericity was used to test the hypothesis that correlation was an identity matrix. No missing data were reported. All statistical analyses were performed using SAS version 9.3 (SAS Institutet Inc., Cary, NC). A significance level of 5% was chosen.

### Research Ethics Committe approval

The committee of Ethics Research at Karolinska Institute in Stockholm approved the study (2008/1929‐31/5).

## Results

Participants’ characteristics are described in Table 1. Women constituted the majority of the respondents (69%). Patients’ mean age was 65 years (sd 8). Most of the participants had only attended the elementary schools, especially among men. Half of the participants were married or living with a partner. The majority (73%) had been diagnosed with moderate COPD [forced expiratory volume (FVC) at 1 second <60%] less than 3 years before. Participants had smoked for an average of 45 years and had an average of three past quit attempts (sd 4). Daily smokers consumed an average of 15 cigarettes per day (sd 7). All of the 63 respondents who were in the process of quitting (right‐hand side of Table 1) were included in the present assessment of TTQ. The demographic characteristics of this group reflected those of the whole sample except for education and for FEV_1_%.

**Table 1:** Demographic and behavioural characteristics of the participant.

Characteristic	All respondents (*n *=* *102)				Respondents trying to quit smoking (*n *=* *63)			
	Total	Male	Female	*P* value	Total	Male	Female	*P* value
*N* (%)	102	32 (31·4)	70 (68·6)	0·00	63	19 (30·2)	44 (69·8)	0·00
Mean age (sd)	64·9 (7·8)	62·3 (7·6)	66·1 (7·7)	0·00	65·0	62·3 (8·1)	66·1 (7·9)	0·08
Education (%)								
Elementary school	46 (45·1)	16 (50·0)	30 (42·9)	0·81	25 (39·7)	10 (52·6)	15 (34·1)	0·38
Upper secondary school	43 (42·2)	12 (37·5)	31 (44·3)		33 (52·4)	8 (42·1)	25 (56·8)	
University	12 (11·8)	4 (12·5)	8 (11·4)		5 (7·9)	1 (5·3)	4 (9·1)	
Other	1 (1·0)	0 (0)	1 (1·4)					
Civil status								
Living alone	46 (45·1)	15 (46·9)	31 (44·3)	0·31	30 (47·6)	8 (42·1)	22 (50·0)	0·57
Married/living together	55 (53·9)	16 (50·0)	39 (55·7)		33 (52·4)	11 (57·9)	22 (50·0)	
Time from diagnosis of COPD (column %)								
< 3 year	74 (72·6)	26 (81·3)	48 (68·6)	0·18	48 (76·2)	16 (84·2)	32 (72·7)	0·32
≥ 3 year	28 (27·5)	6 (18·8)	22 (31·4)		15 (23·8)	3 (15·8)	12 (27·3)	
Forced exploratory volume in 1 second (FEV_1_) (column %)								
<30	18 (17·7)	7 (21·9)	11 (15·7)	0·90	15 (23·81)	4 (21·1)	11 (25·0)	0·66
30–50	17 (16·7)	5 (15·6)	12 (17·1)		11 (17·5)	5 (26·3)	6 (13·6)	
50–80	60 (58·8)	18 (56·3)	42 (60·0)		32 (50·8)	9 (47·4)	23 (52·3)	
80–	7 (6·9)	2 (6·3)	5 (7·1)		5 (7·9)	1 (5·3)	4 (9·1)	
Smoking habits (column %)								
Daily smoker	95 (93·1)	31 (96·9)	64 (91·4)		57 (90·5)	18 (94·7)	39 (88·6)	0·44
Weekly smoker	7 (6·9)	1 (3·1)	6 (8·6)		6 (9·5)	1 (5·7)	5 (11·4)	
Cigarette smoking								
Mean cigarettes per day (range)	39 (1–40)	26 (4–30)	39 (1–40)		28 (2–30)	16 (4–20)	28 (2–30)	
Mean cigarettes per week (range)	24 (1–25)	0 (20–20)	24 (1–25)		46 (24–70)	0 (20–20)	24 (1–25)	
Duration of smoking in years, mean (sd)	45 (8·3)	44·1 (8·1)	45·5 (8·4)		45·2 (9·0)	43 (9·7)	46·3 (8·6)	
Numbers of quit attempts, mean (sd)	3·1 (4·2)	3·6 (5·5)	2·8 (3·5)		3·3 (4·3)	3·7 (5·4)	3·2 (3·8)	
Other smokers in the social environment (column %)								
Yes	61 (59·8)	12 (37·5)	29 (41·4)	0·70	30 (47·6)	9 (47·4)	21 (47·7)	
No	41 (40·2)	20 (62·5)	41 (58·6)		33 (52·4)	10 (52·6)	23 (52·3)	

### Exploratory factor analysis (EFA)

Sixty‐three respondents actively trying to quit smoking answered the TTQ's 17 items intended to explore the mental states arising in the process of quitting (Tables 2 and 3) and were included in the EFA. Three factors accounted together for more than 80% of the variance. The Kaiser–Meyer–Olkin (KMO) was 60% and Cronbach's alpha >70% (Table 3). After rotated exploratory factor analysis, three items were excluded because of low loadings (below 0·30) and high uniqueness (>0·70) (Table 2). KMO estimate 60% of the remaining 14 items and the Bartlett test was statistically significant (*P* < 0·00). Analyses resulted in a three‐factor solution including 14 items that accounted together for more than 80% of the variance (Table 3).

**Table 2:** TTQ items, hypothesized factors and factor loading in exploratory factor analysis (EFA).

Item	Hypothesized factor in the theoretical model	Factor loading in EFA
1. I feel criticized for not being able to quit smoking	A	A
2. I criticize myself for not being able to quit	A	A
3. I do not get support and encouragement when I try to quit smoking	A	C
4. I feel worried about consequences if I do not quit smoking	A	Excluded
5. I am worried about physical reactions if I quit smoking	A	C
6. I feel that I must quit smoking	A	C
7. I constantly think about quitting smoking	A	A
8. I perceive it as a failure not being able to quit smoking	A	A
9. I am keen to try new methods as aid to smoking cessation	D	C
10. I do not want information about the progression of COPD	B	C
11. I do not want to show that I smoke	B	A
12. I have difficulties to stop smoking because my husband/wife/friend smokes	B	Excluded
13. It is unnecessary to quit because my health will not improve	B	B
14. It is unnecessary to quit because I am too old	B	B
15. It is unnecessary to quit because decreasing the number of cigarettes is sufficient	B	B
16. I hope to be able to quit smoking some day	E	Excluded
17. I do *not* feel that to quit smoking is meaningful	F	B

A = Developing pressure‐filled mental states; B = Using destructive pressure‐relief strategies; C = Ambivalent thoughts; D = Using constructive pressure‐relief strategies; E = Continue to try; F = Giving up trying.

**Table 3:** Exploratory factor analysis varimax rotation factor loading (n = 63).

Item	Rotated factor pattern (1)					Revised factor pattern (2)				
	F1	F2	F3	U^a^	KMO^b^	F1	F2	F3	U^a^	KMO^b^
1.	**0·37**	0·00	0·19	0·80	0·60	**0·42**	−0·04	0·17	0·77	0·63
2	**0·70**	0·02	0·05	0·50	0·71	**0·72**	0·00	0·04	0·48	0·73
3	−0·20	0·22	**0·42**	0·74	0·44	−0·16	0·24	**0·40**	0·76	0·39
4	0·33	−0·04	−0·02	0·89	0·36	–	–	–	–	
5	−0·06	0·03	**0·66**	0·57	0·53	−0·02	0·04	**0·65**	0·58	0·50
6	0·41	−0·18	**0·47**	0·50	0·70	0·40	−0·14	**0·44**	0·58	0·75
7	**0·71**	0·10	0·22	0·39	0·70	**0·72**	0·11	0·21	0·39	0·66
8	**0·63**	−0·02	−0·02	0·61	0·71	**0·65**	−0·05	0·02	0·57	0·72
9	−0·31	−0·02	−**0·46**	0·64	0·72	−0·33	−0·04	−**0·47**	0·62	0·70
10	−0·04	0·08	−**0·41**	0·82	0·50	−0·08	0·04	−**0·41**	0·82	0·46
11	**0·58**	0·21	−0·25	0·64	0·50	**0·55**	0·20	−0·30	0·64	0·52
12	0·03	0·26	0·33	0·81	0·58	–	–	–	–	
13	0·22	**0·62**	0·12	0·55	0·58	0·20	**0·66**	0·06	0·52	0·68
14	0·07	**0·76**	0·09	0·41	0·50	0·06	**0·77**	0·04	0·39	0·54
15	0·08	**0·86**	−0·18	0·26	0·50	0·03	**0·78**	−0·17	0·39	0·52
16	−0·24	0·31	−0·21	0·77	0·53	–	–	–	–	
17	−0·33	**0·45**	0·19	0·63	0·50	−0·33	**0·48**	0·17	0·62	0·51
										
										
Eigenvalue (%)	2·98 (0·37)	2·27 (0·28)	1·21 (0·15)			2·73 (0·43)	2·02 (0·32)	1·11 (0·18)		
Variance (%) after rotation	2·69 (0·34)	2·24 (0·28)	1·86 (0·23)			2·50 (0·39)	2·02 (0·32)	1·57 (0·25)		
Overall KMO^b^					0·58^d^					0·60^d^
Overall Cronbach's alpha^c^					0·72					0·71

Bold marked values represent the chosen items in each factor based on uniqueness Gray marked values represent abs (loading) < 40.

a Uniqueness.

b Kaiser–Mayer–Olkin (KMO) measure of sampling adequacy.

c Scale reliability coefficient ‘Cronbach's Alpha Based on Standardized Items’.

d A high significant Bartlett's Test of Sphericity (p < 0·00).

Latent variables were labelled: 1. *Developing pressure‐filled mental states* (F1) while trying to quit smoking, i.e. perception of being criticized, harbouring constant thoughts about quitting and feelings of worthlessness (5 items), (score 11·83 ± 0·63 max score 20 and min 5). 2. *Use of destructive pressure‐relief strategies* (F2) included items indicating the quest for alibi not to proceed to action, such as endorsement of quitting being meaningless because of high age; or cutting down the daily consumption as sufficiently effective strategy (4 items) (score 5·41 ± 0·30 max 16 and min 4). 3. *Ambivalent thoughts* (F3), such as perceiving lack of support in the social environment, fear of physical reactions following abstinence, feeling obliged to quit (positively loading), accepting information about new methods in smoking cessation and refusing information on the progression of the disease (negatively loading) (5 items); (score 11·21 ± 0·40 max 20 min 5) (Table 4).

**Table 4:** Response distribution of TTQ items among COPD patients trying to quit smoking (*n* =63^a^).

Item	Likert scale							
	1 (%)	2 (%)	3 (%)	4 (%)	Mean (se)	Median (sd)	Ranges (Min–Max)	Q1–Q3
1	24 (38·10)	15 (23·81)	7 (11·11)	17 (26·98)	2·27 (0·16)	2 (1·23)	3 (1–4)	(1–4)
2	8 (12·70)	15 (23·81)	9 (14·29)	31 (49·21)	3·00 (0·14)	3 (1·12)	3 (1–4)	(2–4)
3	42 (66·67)	12 (19·05)	6 (9·52)	3 (4·76)	1·52 (0·11)	1 (0·86)	3 (1–4)	(1–2)
4	5 (7·94)	11 (17·46)	8 (12·70)	39 (61·90)	3·29 (0·13)	4 (1·02)	3 (1–4)	(2–4)
5^a^	27 (42·86)	15 (23·81)	9 (14·29)	11 (17·46)	2·06 (0·15)	2 (1·14)	3 (1–4)	(1–4)
6	11 (17·46)	14 (22·22)	12 (19·05)	26 (41·27)	2·84 (0·15)	3 (1·15)	3 (1–4)	(2–4)
7	11 (17·46)	16 (25·40)	10 (15·87)	26 (41·27)	2·81 (0·15)	3 (1·16)	3 (1–4)	(2–4)
8	20 (31·75)	11 (17·46)	10 (15·87)	22 (34·92)	2·54 (0·16)	3 (1·27)	3 (1–4)	(1–4)
9^b^	26 (41·27)	5 (7·94)	12 (19·05)	20 (31·75)	2·41 (0·17)	3 (1·32)	3 (1–4)	(1–4)
10	39 (61·90)	10 (15·87)		14 (22·22)	1·83 (0·15)	1 (1·23)	3 (1–4)	(1–2)
11	37 (58·73)	15 (23·81)	4 (6·35)	7 (11·11)	1·70 (0·13)	1 (1·01)	3 (1–4)	(1–2)
12	55 (87·30)	3 (4·76)	1 (1·59)	4 (6·35)	1·27 (0·10)	1 (0·79)	3 (1–4)	(1–1)
13	56 (88·89)	6 (9·52)	1 (1·59)		1·13 (0·15)	1 (0·38)	2 (1–3)	(1–1)
14	52 (82·54)	7 (11·11)	2 (3·17)	2 (3·17)	1·27 (0·19)	1 (0·68)	3 (1–4)	(1–1)
15	52 (82·54)	8 (12·70)	1 (1·59)	2 (3·17)	1·25 (0·18)	1 (0·65)	3 (1–4)	(1–1)
16^b^	60 (95·24)	1 (1·59)	2 (3·17)		1·08 (0·15)	1 (0·37)	2 (1–3)	(1–1)
17	49 (77·78)	9 (14·29)	2 (3·17)	3 (4·76)	1·35 (0·10)	1 (0·77)	3 (1–4)	(1–1)

a Item 5 one missing respondent.

b Item responses revised in EFA.

Q1, Lower Quartile Q3, Upper Quartile; Likert scale 1 = do not agree to 4 = do agree completely).

Items included in factor 1 developing pressure‐filled mental states scores from mean 1·70–3·00 (max 4·00). There was tendency of a floor effect in the response profile in factor 2 using destructive pressure‐relief strategies, mean score from 1·13–1·35 (max 4·00). For factor 3 ambivalent thoughts the item score from mean 1·52–2·84 (max 4·00) (Table 4).

We also found that women's score on pressure‐filled mental states was significantly higher than men, 13·30 (3·9) vs. 10·05 (3·5), *P* value <0·00. There were no significant differences between women and men in use of destructive relief strategies 4·89 (1·9) vs. 5·3 (1·7), *P* value <0·46 and ambivalent thoughts 10·65 (2·2) vs. 10·79 (2·4), *P* value <0·82.

## Discussion

### Main findings

This paper reports on the psychometric properties of a novel instrument, the TTQ questionnaire, based on a theoretical model applied to COPD smokers planning a quit attempt. Of the original 17 items included in the scale, 14 items were retained in the factor analyses and loaded on three factors, indicating: (1) development of pressure‐filled mental states (5 items explaining different feelings associated with low self‐efficacy and efforts to conceal smoking when not succeeding); (2) use of destructive pressure‐relief strategies (4 items explaining strategies of rationalizing); and (3) ambivalent thoughts (5 items related to contrasting attitudes towards quitting). These items performed well on test of KMO and had high internal consistency. An exploratory factor analysis indicated that these three factors explained over 80% of variance in the data.

The face validity of the TTQ (i.e. adherence to the underlying theoretical model) seemed partial (Lundh *et al*. 2012). Two of the factors identified by factor analysis corresponded rather well with those hypothesized by the theoretical model, that postulated pressure‐filled mental states and destructive pressure‐relief strategies factors, leading to hopelessness and resignation, therefore to continued smoking (Lundh *et al*. 2012). However, two of the originally hypothesized items did load on other factors, i.e. ‘concealing smoking status’, that loaded on the factor indicating ‘pressure‐filled mental state’ and fear of getting information on disease progression, that loaded on a third factor identified as ‘ambivalent thoughts’. This latter factor was not identified as a predictor of smoking cessation in the theoretical model. Items loading on this factor were originally used to indicate either pressure‐filled mental states or use of constructive pressure‐relief strategies. This discrepancy may indicate insufficiency of the brief assessment instrument TTQ towards predictors of both unsuccessful and successful quitting (constructive strategies) and/or failure of the theoretical model to include important mental processes such as ambivalent cognition. It was also found that women were more prone to develop pressure‐filled mental states than men. This is in agreement with studies reporting that women experience poorer health‐related quality of life than men with COPD and also show more frequent occurrence of anxiety and depression (Naberan *et al*. 2012). Therefore, TTQ can be a useful guidance in tailoring counselling on women's need.

### Strengths and limitations of this study

The purpose of this study was to investigate if the novel theory‐based instrument TTQ could reliably measure mental processes involved in smoking cessation among people with a diagnosis of COPD. Strengths of the study include the usual care setting that allowed the simultaneous exploration of feasibility, management and performance of the instrument. Several limitations may have impaired the ability to detect a deeper correspondence between the theoretical model underpinning the instrument and the empirical findings, above all the small sample size. Further, we were not able to study construct validity of the TTQ instrument at this stage. To do this, besides a larger sample, one would need an external reference (e.g. correlation with scales measuring the same factors) that was lacking in this study.

In line with the natural history of the disease, participants were mostly older, moderate to heavy smokers, with an average duration of smoking of 45 years. Furthermore, the sample consisted mostly of women, in agreement with the higher prevalence of COPD among Swedish women than men (GOLD 2013, National Board of Health & Welfare 2013). Other selection factors (i.e. at the clinic level) may also have been in place. Therefore, caution is needed when generalizing findings and conclusions to the whole population of smokers with COPD. However, as the nurses did not report any refusal to participate, participants should be fairly representative of the patient population served by primary care facilities in Sweden.

The TTQ was developed to identify pressure‐filled mental states and destructive strategies when trying to quit smoking specifically among patients with COPD; therefore, its usefulness among smokers with other diseases or in non‐clinical settings cannot be assumed. Also, satisfactory content and face validity do not imply actual usefulness of the instrument in predicting the risk of failure in a given quit attempt among COPD patients, an aspect that remains to be investigated in the frame of a longitudinal study.

### Interpretations of findings in relation to previously published work

Other studies have found that anticipated negative self‐evaluative emotions, defined as being dissatisfied with own smoking, feeling stupid, fed up, guilty, ashamed, in relation to behaviour change were connected with higher risk of relapses into smoking (Dijkstra & den Dijker 2005, Menninga *et al*. 2011). Therefore, it can be of importance to identify persons with high feeling of pressure‐filled mental states and use of destructive pressure‐relief strategies.

Most of the destructive pressure‐relief strategies described in the theoretical model also loaded on the same factor in the EFA. Perceiving ambivalence is an unavoidable component of all behavioural changes, as a part of the discrepancy between pros and cons in a decision, such as quitting smoking. Low ambivalence can indicate high motivation to quit smoking (Prochaska *et al*. 1992). On the other hand, if ambivalence is still present after decision about quit smoking is made, there is an increased risk for relapse (Baillie *et al*. 1995, Menninga *et al*. 2011). The relation of perceived ambivalence with relapse can also be mediated by triggers connected to situations where smoking was habitual in the past (Menninga *et al*. 2011). This indicates that patients’ ambivalence may be an important aspect to be considered in relapse prevention. If so, understanding ambivalence would probably be major cue in motivational counselling (Balmford & Borland 2008).

The usefulness of TTQ rests on its ability to measure determinants of protracted smoking not necessarily corresponding to a tobacco dependence syndrome. Indeed, the instrument deals with feelings of pressure‐filled mental states, for instance negative criticism and worthlessness. Destructive relief strategies, leading to giving up further quit attempts, specific to patients with COPD, are also tackled by the instrument, something that was not assessed or evaluated in previous studies.

Other instruments have attempted to include emotional aspects of the process of quitting smoking. For instance, a well‐known clinical instrument, the 55‐item Smoking Abstinence Questionnaire (SAQ), aims to measure the role of abstinence‐related expectancies, such as withdrawal, social improvement/non‐smoker identity, adverse outcomes, common reasons to quit smoking, optimistic outcomes (Hendricks *et al*. 2011). The 12‐item Cigarette dependence scale (CDS) measures both nicotine dependence and some emotional aspects of the process of quitting smoking (5).

On the same track, the Smoking Abstinence Self‐Efficacy Questionnaire (SASEQ) is a 6‐item questionnaire measuring two dimensions describing social and emotional situations in risk of relapse. People who quit smoking had significantly higher scores of SASEQ before quitting than those who were not motivated to quit smoking (Speak *et al*. 2013).

Finally, the Brief Smoking Consequences Questionnaire – Adult (BSCQ‐A) was developed to assess smoking outcome in adult heavy smokers according to their expectancies towards smoking or smoking cessation. Outcome expectancies are assessed on 10 scales named negative affect reduction, stimulation/state enhancement, health risk, taste/sensorimotor manipulation, social facilitation, weight control, craving/addiction, negative physical feelings, boredom reduction and negative social impression (Rash & Copeland 2008).

Some of these scales are of interest because they can be easily combined with TTQ for in‐depth assessment of need for support, as they cover both overlapping and different constructs. For instance, items in the CDS (Etter *et al*. 2003), such as the feeling to be prisoner of cigarettes, seem to relate well to pressure‐filled mental states assessed by the TTQ. Items in the SAQ (Hendricks *et al*. 2011) assessing expectations about withdrawal and adverse reactions when quitting are closely related to TTQ's pressure‐filled mental states and ambivalent thoughts. As TTQ assesses feelings and thoughts not associated with self‐efficacy, it can be a useful complement to the SASEQ scale (Speak *et al*. 2013) in predicting successful smoking cessation. Negative social impressions in the BSCQ‐A (Rash & Copeland 2008) can be related to pressure‐filled mental states and feelings of criticism.

## Conclusions

The new assessment instrument ‘Trying to quit smoking’ (TTQ) can be useful to predict factors that have a negative impact on attempts to quit smoking among patients with COPD and guide professional support to smoking cessation.

### Relevance to clinical practice

The TTQ instrument reliably identifies factors such as high degree of pressure‐filled mental states, use of destructive pressure‐relief strategies and feelings of ambivalent thoughts among patients with COPD. Patients with high scores in these domains, i.e. high feelings of pressure‐filled mental states, may need tailored individual advice to succeed in their attempts to quit smoking. TTQ is a short instrument that easily identifies these patients in a clinical setting, allowing among other things a more efficient use of the nurses’ time. In conclusion, the TTQ instrument can represent a useful tool to healthcare providers when counselling COPD patients for smoking cessation.

## Conflict of interest

The authors declare that they have no conflicts of interest in relation to this article.

## Author contributions

LL, LT, HG and MRG participated in the concept and design of the study. All authors participated in the design of the analysis plan and the interpretation of the data. LL, HA, LT and RMG analysed the data and drafted the manuscript. LT, HG and RMG critically revised the manuscript and supervised LL. All authors read and approved the final manuscript.

All authors have agreed on the final version and meet at least one of the following criteria [recommended by the ICMJE (http://www.icmje.org/ethical_1author.html)]:

- substantial contributions to conception and design, acquisition of data, or analysis and interpretation of data;
- drafting the article or revising it critically for important intellectual content.
